# Impact of Indirect Trauma and Disaster Media Exposure on Psychological States and Temporal Processes: The Case of 2023 Turkey Earthquakes

**DOI:** 10.1002/cpp.70008

**Published:** 2024-11-21

**Authors:** Irem Tugce Oz, Giorgia Cona

**Affiliations:** ^1^ Department of General Psychology University of Padova Padova Italy; ^2^ Louvain Experimental Psychopathology Research Group (LEP) Psychological Sciences Research Institute (IPSY), UCLouvain Louvain‐La‐Neuve Belgium; ^3^ Padua Neuroscience Centre University of Padova Padova Italy

**Keywords:** delay discounting, earthquake, indirect exposure, PTSD, trauma

## Abstract

Turkey experienced two devastating earthquakes, which resulted in more than 50,000 deaths and millions of injured and homeless individuals. The negative influence of direct exposure to trauma has been proven, but the impact of indirect exposure remains unclear. In this study, we focused on indirect types of trauma exposure: the loss of someone in the earthquake and the exposure to disaster media. We aimed to explore the influence of these dimensions on psychological states, including earthquake trauma severity, post‐traumatic stress disorder (PTSD) symptoms, depression, anxiety, hopelessness and life satisfaction and temporal‐psychological measures, such as intertemporal decision‐making tendencies and time perspectives. The sample (*N* = 215) consisted of Turkish individuals who experienced the earthquakes through two types of indirect exposure: having lost someone and being exposed to disaster media. Findings showed that having a loss in the earthquake was related to high levels of trauma, anxiety, hopelessness and a past‐negative time perspective. Repetitive exposure to disaster media was linked to higher levels of trauma, PTSD symptoms, depression, anxiety, stress and a past‐negative time perspective. Importantly, the amount of traumatization in case of losing someone was modulated by the frequency of disaster‐media exposure. Even indirect exposure to the disaster substantially disturbs many processes, and the media magnifies such disturbances.

**Summary**

- There is a vital need for easily accessible psychological aid following an earthquake trauma not only for the victims but also for the people who have been exposed to earthquakes in indirect ways (e.g., loss of someone and being exposed to disaster‐related media).
- Indirect trauma exposure has a profound effect on mental health as well as temporal processes, such as overemphasizing the negative view of the past, which points out the importance of psychological assessment for the whole population experiencing the disaster in different ways.
- Repetitive exposure to disaster media has a critical role in exacerbating the impact of indirect trauma on mental health. Hence, media usage during disaster times should be limited to prevent future psychopathology.
- In the current technological era, traumatic scenes are brought directly into households, potentially affecting everyone. Therefore, when defining trauma, future versions of the DSM might consider the impact of media on mental health during disasters.
- In countries where devastating disasters are common due to geography, post‐traumatic assessment should target all populations and qualify for preventive mental health services. Governments should prioritize psychological recovery as much as physical recovery.

## 1 Introduction

In February 2023, Turkey experienced the two most devastating earthquakes in its history, with magnitudes of 7.7 and 7.6. These earthquakes resulted in over 50,783 fatalities, which affected approximately 13.5 million people (BBC News 2023). Natural disasters such as earthquakes are known to lead to unanticipated devastation to the environment, as well as injury, death and financial difficulties for the survivors, which can all lead to trauma (Goenjian et al. 2011). All of these conditions constitute risk factors for individuals who have directly or indirectly experienced trauma, increasing the likelihood of developing symptoms of post‐traumatic stress disorder (PTSD), anxiety and substance abuse (Başoğlu, Salcioglu, and Livanou 2002; Bonanno et al. 2010; Jiang et al. 2016). In addition to their impact on psychological status, traumatic experiences are linked to changes in temporal processes such as intertemporal decision‐making (Matsuyama et al. 2020) and may be linked to how one ‘locates’ him/herself in time. The current study aimed to explore the post‐traumatic situation in the Turkish population from a variety of psychological facets that can be clustered under the name of two constructs: psychological states and temporal‐psychological processes.

To understand what the definition of trauma is, we must investigate the PTSD criteria in DSM‐V. The diagnostic criteria for PTSD in DSM‐V are defined as follows: ‘Exposure to actual or threatened death, serious injury, or sexual violence in one (or more) of the following ways: 1) Directly experiencing the traumatic event(s); 2) Witnessing, in person, the event(s) as it occurred to others; 3) Learning that the traumatic event(s) occurred to a close family member or close friend. In cases of actual or threatened death of a family member or friend, the event(s) must have been violent or accidental; 4) Experiencing repeated or extreme exposure to aversive details of the traumatic event(s) (e.g., first responders collecting human remains; police officers repeatedly exposed to details of child abuse)’. The 4° bullet in the criteria continues with a note stating ‘Criterion A4 does not apply to exposure through electronic media, television, movies, or pictures unless this exposure is work‐related’ (American Psychological Association [APA] 2013). Hence, to be defined as trauma in PTSD, there should be either a direct experience (first‐hand) or an indirect experience by witnessing someone else experiencing the traumatic event or being exposed to traumatic media content repeatedly (indirectly) (May and Wisco 2016). What raises attention is the media exposure criteria in DSM‐V, namely, the statement that the repeated exposure to negative views of the traumatic event via media is limited to professionals (APA 2013). However, the existing literature and the current research proposed that even in the absence of direct contact with the traumatic event, an indirect exposure—such as being exposed to disaster media (Pfefferbaum et al. 2014; Thompson et al. 2019) or loss of someone (Gao et al. 2019)—might influence the psychological well‐being and temporal‐psychological processes. Taking the DSM‐V definition into account, the current study investigated two types of indirect exposure: the loss of someone (criteria 3) and the exposure to disaster media content (Criteria 4). Although the latter is considered as trauma only in case it is professional‐related, in the current sample, we investigated such trauma also in general population, who were not professionals but were still exposed to disaster media repeatedly. Trauma‐related media exposure was associated with distress via indirect trauma (Ben‐Zur, Gil, and Shamshins 2012). Furthermore, traumatic experiences influence psychological well‐being such as depression (Ehring, Razik, and Emmelkamp 2011), post‐traumatic stress symptoms (Neria, Nandi, and Galea 2008), anxiety (Bonanno 2004) and hopelessness (Scher and Resick 2005). In addition to the impact of indirect trauma exposure on mental health, it also raises the question of whether it might be related to changes in temporal‐psychological processes, such as time perspective (i.e., how we think ourselves in relation to past, present and future) and intertemporal decision‐making. Despite several studies revealing the effects of direct or indirect traumatic experience of an earthquake on psychology and decision‐making (Matsuyama et al. 2020), it still needs further exploration of how an earthquake affects the people who were exposed to the traumatic event purely indirectly (without any direct contact with the trauma), specifically via a loss or disaster‐related media.

It is crucial to study the post‐traumatic psychological situation in the whole population since trauma can lead to collective distress that can result in psychopathological symptoms, such as PTSD or depression. Although the lifelong prevalence of PTSD was reported as 6.8%, it may escalate to as high as 11% within the population following a disaster (Galea, Nandi, and Vlahov 2005). Notably, the risk of developing PTSD is not limited to direct but also indirect exposure via media (Neria and Sullivan 2011). On the other hand, depressiveness may increase following trauma. In particular, losing a close loved one, and going through bereavement, is considered a significant risk factor for developing long‐term depression (Gao et al. 2019). Thus, indirect exposure via an earthquake‐related loss or disaster media might be a risk factor for developing PTSD or depression symptoms. However, this was studied on people who directly experienced the earthquake; hence, it is still unknown how loss impacts depressive symptoms without any direct contact with the trauma, which is one of the explorations of the present study.

Greater exposure to disaster media was associated with increased post‐traumatic psychological reactions (Bonanno et al. 2010), including heightened levels of post‐traumatic stress, depression and emotional distress (Houston 2009). A study demonstrated that, during the COVID‐19 lockdown, overexposure to COVID‐19‐media amplified distress, anxiety and depression (Fiorenzato et al. 2021). Moreover, reviews and articles emphasized the negative impact of disaster media exposure (DME) on mental health for people who have been exposed to trauma indirectly (Houston, Spialek, and First 2018; Pfefferbaum et al. 2014; Thompson et al. 2019). Even though DME is not considered a criterion for PTSD, it is linked with PTSD (Hall et al. 2019). Media might be seen as a gateway to relieve the anxiety of uncertainty while carrying the risk of creating a collective trauma (Hall et al. 2019). On the contrary, some scholars found that social media was a collective tool to overcome the disruption of the disaster (Tandoc and Takahashi 2017), for example, heroic images and videos (e.g., rescuing a victim) (Hall et al. 2019). Despite the well‐documented negative influence of DME in the literature, the opposite view is also supported (Hall et al. 2019; Tandoc and Takahashi 2017). Hence, the present study aimed to add to the literature by exploring the effect of DME on temporal‐psychological processes as well as mental health and—more specifically—in individuals who did not directly experience the trauma but were exposed in different indirect ways. Thus, the severity of depression, anxiety and stress in individuals who experienced trauma in different ways is essential to prevent future psychopathology.

Other psychological states such as life satisfaction and hopelessness are found to be linked with post‐traumatology. Previous studies established the relationship between exposure to trauma and lower levels of life satisfaction for both direct (Şeker 2016; Triplett et al. 2012) and indirect trauma (Chang and Taormina 2011; Oishi, Kohlbacher, and Choi 2018). Few studies to date have examined the level of life satisfaction after a traumatic experience in different indirect exposure types. Hopelessness, on the other hand, was rarely studied in the general population following trauma. Notably, the level of hopelessness and PTSD symptoms were correlated (Ozdemir et al. 2015). However, it remains unclear the impact of indirect trauma on hopelessness. Traumatic experiences can significantly impact hopelessness, particularly about various types of exposure, which makes it an important point to explore in case of earthquake trauma.

In addition to exploring the psychological states following trauma, the current study aimed to investigate the effect of the indirect experience of an earthquake trauma on two temporal dimensions: intertemporal decision‐making and time perspective. Intertemporal decision‐making refers to the process of making decisions that involve trade‐offs between gains or losses at different points in time (Frederick, Loewenstein, and O'Donoghue 2002). The typical task is the delay discounting task (DDT), in which people are asked to choose between a sooner but smaller gain and a later but larger gain. Though in fact, the economic value of the later but larger gain is bigger than that of the sooner but smaller gain, individuals might tend to assign a lower subjective value to the later but larger gain due to the time delay to reach that gain, which results in the choices of immediate but smaller rewards, defined as delay discounting (DD) (Bickel and Marsch 2001; Green and Myerson 1996). Examples of DD in daily life can be observed when individuals choose to forgo saving money for retirement in favour of immediate gratification, such as spending money on luxury items or entertainment. DD is associated with excessive alcohol consumption (Bjork et al. 2004), gambling (Reynolds 2006), major depressive disorder (Amlung et al. 2019) and higher levels of depression, anxiety and stress (Cona et al. 2019). Disasters are found to be influential in cognitive processes such as decision‐making (Sacco, Galletto, and Blanzieri 2003). The period after a disaster is full of uncertainty (Houston, Spialek, and First 2018), and when individuals are under uncertain conditions, their decision‐making tendencies change (Kahneman and Tversky 2013). As the levels of stress increase, delay discounting also increases (Fields, Ramos, and Reynolds 2015). Hence, post‐traumatic stress might play an essential role in shaping delay discounting tendencies, where individuals may choose sooner but smaller rewards instead of waiting for a larger reward. This argument raises the issue of how traumatic experiences impact DD tendencies, leading however to mixed results. Several studies showed that after a traumatic experience, individuals tend to prefer sooner but smaller gains instead of later but larger gains (Bryan and Bryan 2021; Li, Li, and Liu 2011; van den Berk‐Clark et al. 2018). On the other hand, another study showed that in the trauma context, DD tendency was not affected by PTSD severity (Olin et al. 2022). Importantly, all these studies focused on the impact of direct exposure. To our knowledge, indeed, no study has investigated the effect of indirect trauma experience on DD, where the present study aimed to fill this gap.

The other temporal‐psychological process is the so‐called ‘time perspective’. Time perspective is the process of assigning values to the past, present and future and happens unconsciously (Košťál et al. 2016). An out‐of‐balanced time perspective like focusing only on the present gains or overemphasizing the negative experiences in the past is an obstacle to healthy functioning; therefore, re‐balancing these temporal biases should be a priority to opt for healthier decisions (Boniwell and Zimbardo 2003). Zimbardo and Boyd (1999) demonstrated a cross‐culturally validated model with five dimensions of time perspective as past‐positive, past‐negative, present‐hedonic, present‐fatalistic and future. The past‐positive represents pleasant and warm feelings and memories regarding the past, whereas the past‐negative reflects an unpleasant and regretful attitude toward the past. The present‐hedonistic view shows a tendency to take risks in an impulsive way to achieve pleasure immediately and is associated with impulsivity (Koós et al. 2022). On the other hand, present‐fatalistic has a desperate view of the present where individuals do not perceive themselves as in control of what they do. Lastly, future is linked with making decisions based on future outcomes, such as desires and goals. Past‐positive‐oriented people were more likely to have lower levels of depression, whereas present‐fatalistic orientation was associated with higher levels of anxiety and depression (Micillo et al. 2022). Contextual factors can have an impact on time perspective (Zimbardo and Boyd 1999), such as having an overvalued past‐negative and present‐fatalistic following war trauma (Senyk et al. 2022). The current study aimed to provide insights about time perspective following indirect trauma exposure, to explore if any dimension of the time perspective is overemphasized following an indirect earthquake trauma and to enlighten the way to encourage including time perspective balancing practices in the therapeutic interventions. In addition to explain the post‐traumatic mental health in indirectly affected populations, the more important contribution of the present study is to understand the post‐traumatic change in temporal‐psychological processes, which was rarely studied.

To sum up, the current research focused on exploring the psychological states (first aim) and temporal‐psychological processes (second aim), as intertemporal decision‐making and time perspective, in people who lost someone in the earthquake and who have been repeatedly exposed to disaster media during earthquake period. To accomplish these aims, we administered online questionnaires and decision‐making tasks to the Turkish population 2 months after the two earthquakes that devastated Turkey in February 2023. Two cohorts of individuals were studied: individuals who lost someone in the earthquake (vs. those who did not) and individuals who were exposed to the disaster media repeatedly (vs. those who were exposed less). Notably, people who have experienced the earthquakes, hence, had direct exposure but were not included in the two cohorts.

## 2 Methods

### 2.1 Participants

All procedures were approved by the Institutional Review Board of the University of Padua. A total of 410 Turkish individuals participated in the online survey 2 months after the earthquakes between April and May 2023. The sample underwent a selection based on these exclusion criteria: age lower than 18, being exposed to earthquakes directly, using psychiatric/neurological medications and/or having a neurological/psychiatric disorder as some of these pathologies are associated with changes in psychological states and in delay discounting (e.g., major depressive disorder, bipolar disorder, and eating disorders) (Amlung et al. 2019), which can be confounding factors on the variables assessed in the current study. Following the exclusion criteria and dropouts, the final sample consisted of 215 participants. The sample consisted of individuals with an age range from 18 to 64 (*M* = 32.28, *SD* = 12.08), and of 77% females (*N* = 166) and 21% males (*N* = 46), 1% did not want to specify (*N* = 3). Excluded participants were drop‐outs (*N* = 152)—thus people who started but did not complete the questionnaires, individuals with neurological/psychiatric disorders or using medications (antidepressant/antipsychotics) (*N* = 42) or individuals with direct exposure to the earthquake (*N* = 1).

To construct the earthquake‐related loss variable, we asked three questions regarding having a loss, respectively a family member, a friend or an acquaintance. In the case of an answer of ‘yes, I had a loss’ to any of these questions, this was treated as the loss group (*N* = 146). Those who answered ‘no, I did not have a loss’ to any of the three loss questions were the no‐loss group (*N* = 69). For the second variable, namely, DME, a 6‐point Likert scale was used to assess how often participants have been exposed to disaster media. The larger part of the sample has been exposed to disaster media all day long (*N* = 143), which refers to the highest score (6) on the Likert scale. The rest of the sample was exposed to disaster media less, but these groups were not enough to analyse individually. Hence, the rest of the sample was merged to be able to compare the full exposure and less exposure to disaster media (from 1 to 5 Likert scale; *N* = 72) (see Table 1).

**TABLE 1:** Characteristics of the sample.

Variable	Level	Counts	Total	Proportion
Earthquake‐related loss	No	69	215	0.32
	Yes	146	215	0.67
Disaster media exposure	Less exposure	72	215	0.33
	Full exposure	143	215	0.66

*Note:* Proportions tested against value: 0.5.

### 2.2 Instruments

Here, a general description of the instruments is provided; please see Supporting Information for details of the instruments.

#### 2.2.1 Questions Regarding Earthquake Exposure

Following the informed consent and demographic questions, several questions were asked to assess in which way participants were exposed to the earthquakes. These questions were ‘Did you lose a family member in the earthquake?’, ‘Did you lose a friend in the earthquake?’ and ‘Have you heard of the death of an acquaintance?’ for the loss condition. For the DME condition the question was ‘How often were you exposed to the news, videos, and images of the earthquake?’ with a 6‐point Likert scale (6 = *continuous throughout the day*, 5 = *several times during the day*, 4 = *once a day*, 3 = *several times a week*, 2 = *once a week*, 1 = *rarely*).

#### 2.2.2 Scale That Determines the Level of Trauma After the Earthquake

After an earthquake in 2011 in Turkey, a culturally suitable instrument that screens the level of trauma after the earthquake was developed (Tanhan and Kayri 2013). Scholars aimed to create a questionnaire specifically for the post‐traumatic symptoms after an earthquake in Turkey, which matches the present study's goals. The scale had 20 items and each item had 5‐point Likert options (1 = *I do not agree at all* to 5 = *I completely agree*). In this study, the internal consistency of the scale was α = 0.92, whereas in the adaptation of the scale, it was α = 0.87 (Tanhan and Kayri 2013) (see Supporting Information for the details).

#### 2.2.3 PTSD Checklist for Diagnostic and Statistical Manual of Mental Disorders, Fifth Edition (PCL‐5)

PCL‐5 is designed for assessing the severity of the traumatic symptoms after a traumatic experience mapping onto DSM‐V symptoms (APA 2013). PCL‐5 consisted of 20 items (Weathers et al. 2013), with a 5‐point Likert scale (0 = *not at all bothersome* to 4 = *extremely bothersome*). ‘Trouble remembering important parts of the stressful experience?’ is a sample item. The reliability score was α = 0.97 while for the present study, Cronbach's alpha was 0.94 (see Supporting Information for the details).

#### 2.2.4 Depression, Stress and Anxiety Scale (DASS‐21)

The Turkish adaptation of the DASS‐21 by Yılmaz, Boz, and Arslan (2017) was used. DASS‐21 consists of 21 items: seven items per each of the three factors depression, stress and anxiety. The rating was based on a 4‐point Likert scale of 0 = *did not apply to me at all* to 3 = *applied to me very much, or most of the time*. Some items on the scale are listed: ‘I was unable to become enthusiastic about anything’, ‘I was worried about situations in which I might panic and make a fool of myself’ and ‘I couldn't seem to experience any positive feeling at all’. In the current study, Cronbach's alpha for the overall scale was 0.94; and for the subscales, they were 0.91, 0.83 and 0.89, respectively (see Supporting Information for the details).

#### 2.2.5 Satisfaction With Life Scale

Life satisfaction is defined as ‘a global assessment of a person's quality of life according to his chosen criteria’ (Shin and Johnson 1978). The Satisfaction with Life Scale is a self‐report with five items on a 7‐point Likert rating (Diener et al. 1985). During the adaptation of scale into Turkish, the options in the 7‐point Likert were perceived as ‘close to each other’, and to have an easily understandable scale, scholars decreased the number of options to a 5‐point Likert (1 = *strongly disagree* to 5 = *strongly* agree) (α = 0.88) (Dağlı and Baysal 2016). ‘The conditions of my life are excellent’ is an example item. The internal consistency of the scale was 0.87 in the current study (see Supporting Information for the details).

#### 2.2.6 Beck Hopelessness Scale

Hopelessness refers to ‘a lack of enthusiasm, a motivational tendency to give up, and a dearth of positive expectancies’ (Beck et al. 1974). The Beck Hopelessness Scale is used to quantify the level of hopelessness. The Turkish adaptation of the scale had 20 self‐report questions with the answer options as ‘yes’ or ‘no’. Items 1, 3, 5, 6, 8, 10, 13, 15 and 19 were reverse‐coded (Seber et al. 1993). Three factors of the scale were emotional, motivational, and cognitive. ‘I might as well give up because there is nothing I can do about making things better for myself’ is a sample item. Cronbach's alpha of the Turkish validation was 0.86. (Seber et al. 1993), but for this study it was reported as 0.90 (see Supporting Information for the details).

#### 2.2.7 DDT

Following the questionnaires, participants were directed to DDT. In this task, participants were asked to choose between two hypothetical monetary alternatives: an immediate amount—smaller but sooner—(e.g., ₺400.000, now) versus a larger but later amount (e.g., ₺800.000 in 1 year). Six time points such as 1 month, 6 months, 1 year, 3 years, 5 years and 10 years were used. After each choice, the immediate amount was adjusted to identify the amount judged by the subject as equivalent to the delayed amount (i.e., the indifference point). If the participant selected the immediate option, the amount was reduced in the next trial; if they chose the delayed option, the immediate amount was increased. For each time point, participants made five choices. The amount that would have been used in the sixth trial was considered the indifference point, representing the point at which an individual is equally likely to choose a smaller, sooner reward or a larger, later reward. The area under the curve (AUC) was derived calculating the changes of the indifference points as a function of the time points. The AUC ranges from 0 (high discount rate) to 1 (low discount rate). As such, a larger AUC indicates less discounting, whereas a smaller AUC corresponds to greater discounting.

OpenSesame v.3.3.6 (Mathôt, Schreij, and Theeuwes 2012) was used to design the task, and it was run online through JATOS (Lange, Kühnhausen, and Filevich 2015) with the help of OSWeb v.1.3.11 extension.

#### 2.2.8 Zimbardo Time Perspectives Inventory (ZPTI‐15)

This scale aimed to measure the time perspectives, defined as the process of assigning values to the past, present, and future, unconsciously (Košťál et al. 2016). ZPTI‐15 was created by Zimbardo and Boyd (1999) and consisted of 56 items. Later on, it was shortened to 15 items (Košťál et al. 2016). Turkish version of the scale was used, which was rated in a 5‐point Likert scale (1 = *very uncharacteristic* to 5 = *very characteristic*) (Kocayoruk and Simsek 2020). The five subscales were time perspectives as past‐positive, past‐negative, present‐hedonic, present‐fatalistic and future. Some sample items are ‘I think about the bad things that have happened to me in the past’ and ‘It is important to put excitement in my life’. Cronbach's alphas ranged from 0.65 to 0.78 depending on the subscales, whereas in the current study, they ranged from 0.56 to 0.83.

### 2.3 Data Collection Procedure

The online survey is created on Qualtrics with a direct forwarding to JATOS once the questionnaire is completed. Participants filled out the self‐report questionnaires on Qualtrics, and they were directed to JATOS for the DDT. The survey, conducted between April and May 2023, was disseminated across various social media platforms. All responses were kept anonymous, and the survey duration was approximately 10 min. Participation was entirely voluntary, and no incentives were offered to participants.

### 2.4 Data Analysis Procedure

JASP Version 0.17.2 software was used to compute the analysis (JASP 2023). A set of ANCOVAs were run with two independent factors: 2 (earthquake‐related loss: loss/no loss) × 2 (DME: full/less). The dependent variables of the current design are used in each ANCOVA.

Age was considered a covariate in all the analyses to partially out the effect of age. For the significant interactions, additional post hoc analyses were run using Bonferroni correction.

## 3 Results

### 3.1 Psychological States

#### 3.1.1 Earthquake Trauma Severity

**FIGURE 1:**
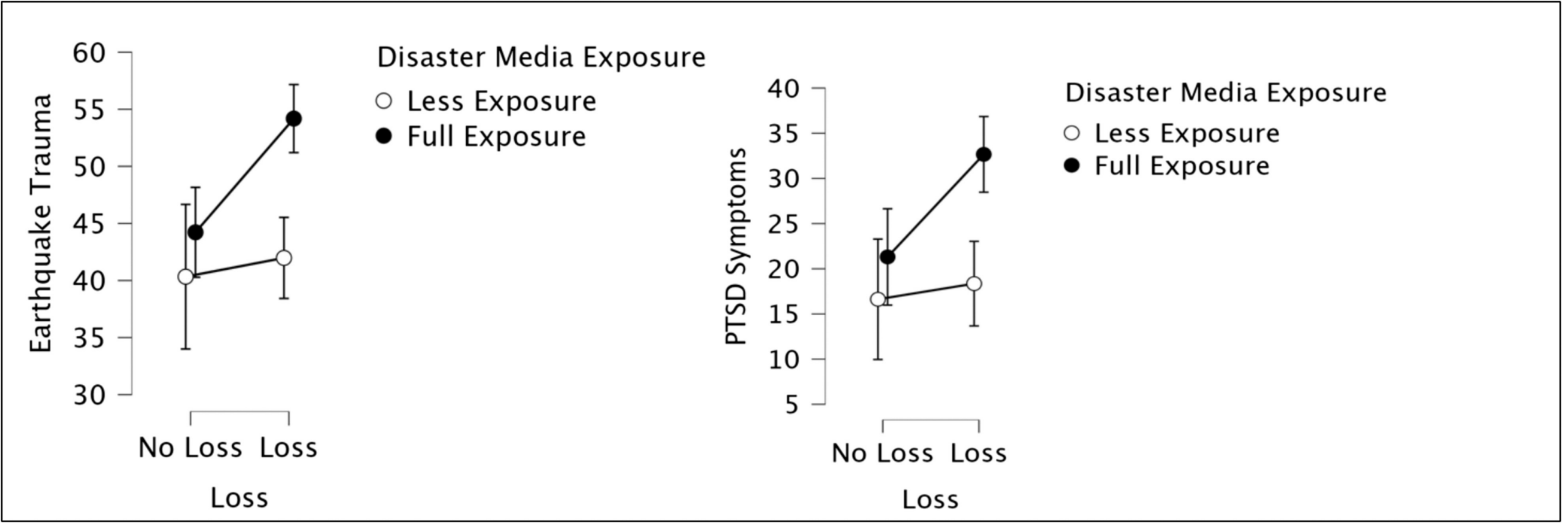
Effects of DME and loss on trauma severity and PTSD.

Analyses showed that there was a significant effect of loss on earthquake trauma severity (*F*_1,210_ = 5.95, *p* = 0.01) in a way that people who lost someone in the earthquakes had significantly more severe levels of earthquake trauma (*M* = 50.17; *SD* = 15.15) as compared to others who did not lose someone (*M* = 42.87; *SD* = 13.81). Also, there was a significant relationship between DME and earthquake trauma severity (*F*_1,210_ = 12.46, *p* < 0.001); participants who were fully exposed to disaster media had significantly more severe earthquake trauma symptoms (*M* = 51.05; *SD* = 15.03) than those who were less exposed (*M* = 41.43; *SD* = 13.13). Furthermore, there was a significant interaction between loss and DME (*F*_1,210_ = 3.93, *p* < 0.05). Importantly, post hoc analyses revealed that participants who had lost someone in the earthquake and were also fully exposed to disaster media had significantly more severe levels of earthquake trauma (*M* = 54.18; *SD* = 14.87) as compared to others who also lost someone but were less exposed to disaster media (*M* = 41.98; *SD* = 12.22, *p* < 0.01). On the other hand, for people who did not lose anyone, there was no difference between people who were more exposed and those less exposed (*p* > 0.05) (see Figure 1).

#### 3.1.2 PTSD Symptoms

Analysis showed that there was no significant relationship between loss and PTSD symptoms (*F*_1,210_ = 2.86, *p* > 0.05). On the contrary, DME and PTSD symptoms were significantly related (*F*_1,210_ = 8.17, *p* = 0.00). People who were fully exposed to disaster media showed higher levels of PTSD symptoms (*M* = 29.09; *SD* = 20.58) than others who were less exposed (*M* = 17.78; *SD* = 15.91). No interaction was found between DME and loss (*F*_1,210_ = 3.40, *p* > 0.05) (see Figure 1).

#### 3.1.3 Depression

**FIGURE 2:**
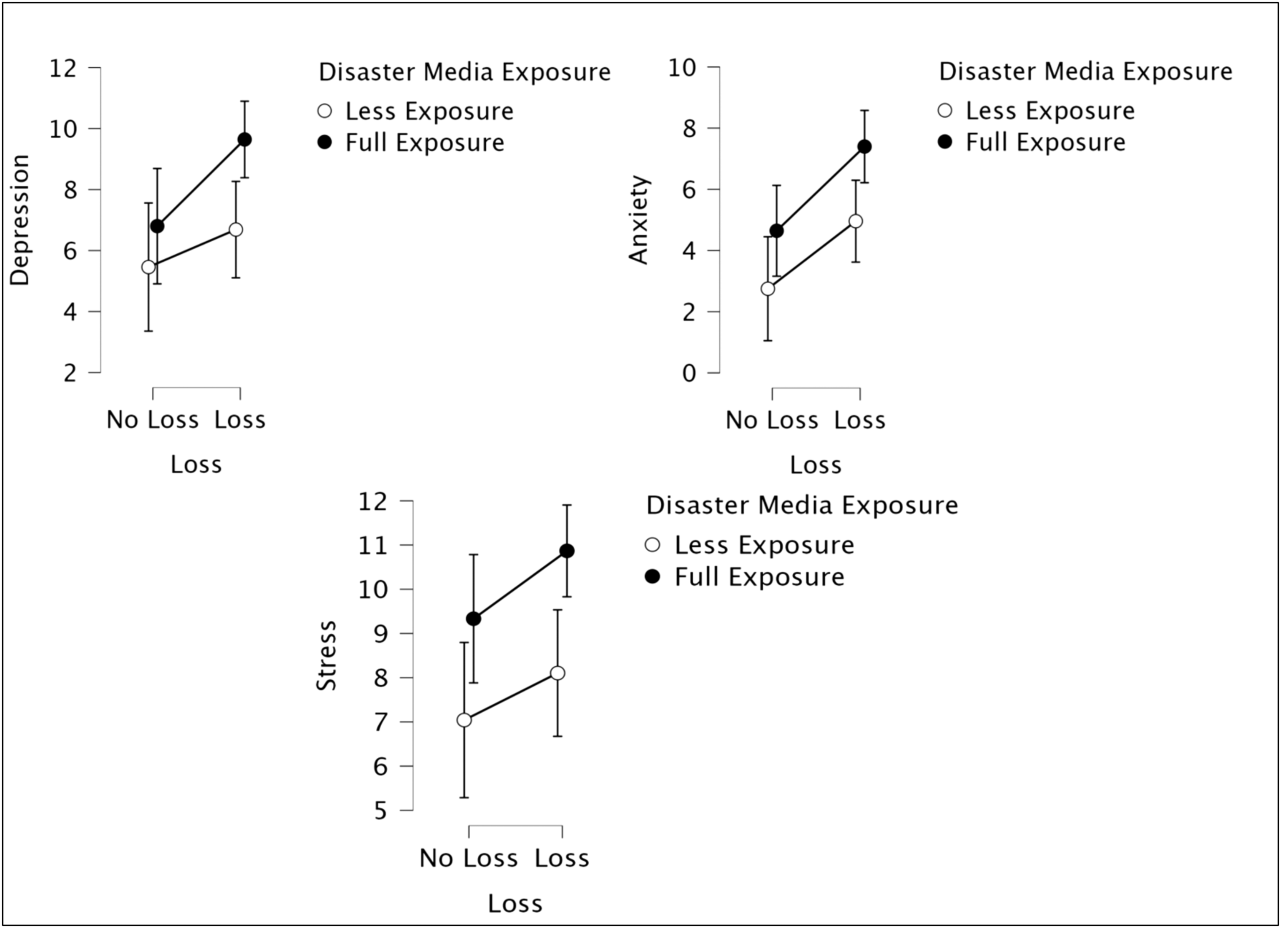
Effects of DME and loss on depression, anxiety and stress.

The results showed that there was no significant link between loss and depression symptoms (*F*_1,210_ = 3.13, *p* > 0.05). However, there was a significant association between DME and depression (*F*_1,210_ = 3.96, *p* < 0.05). Participants who were fully exposed to disaster media had higher levels of depression symptoms (*M* = 8.75; *SD* = 6.38) than participants who were less exposed to earthquake‐related disaster media (*M* = 6.28; *SD* = 5.28). Lastly, there was no interaction between DME and loss (*F*_1,210_ = 0.98, *p* > 0.05) (see Figure 2).

#### 3.1.4 Anxiety

Having a loss in the earthquake and the level of anxiety were significantly associated (*F*_1,210_ = 6.38 *p* = 0.01). Moreover, DME and level of anxiety were significantly related (*F*_1,210_ = 5.12, *p* < 0.05). Individuals who were fully exposed to disaster media showed higher levels of anxiety (*M* = 6.53; *SD* = 5.73) as compared to others who were exposed less (*M* = 4.22; *SD* = 4.51). People who had a loss had higher levels of anxiety (*M* = 6.60; *SD* = 5.60) than those who did not (*M* = 3.99; *SD* = 4.70). There was no interaction effect between DME and loss (*F*_1,210_ = 0.22, *p* > 0.05) (see Figure 2).

#### 3.1.5 Stress

There was no significant relationship between loss and levels of stress (*F*_1,210_ = 1.58, *p* > 0.05). On the contrary, analyses revealed that DME and level of stress were significantly related (*F*_1,210_ = 8.82, *p* = 0.00). Individuals who have been exposed to disaster media repeatedly showed higher levels of stress (*M* = 10.38; *SD* = 5.10) as compared to individuals who have been exposed less (*M* = 7.75; *SD* = 4.68). Lastly, there was no interaction effect of DME and loss (*F*_1,210_ = 0.17, *p* > 0.05) (see Figure 2).

#### 3.1.6 Hopelessness

**FIGURE 3:**
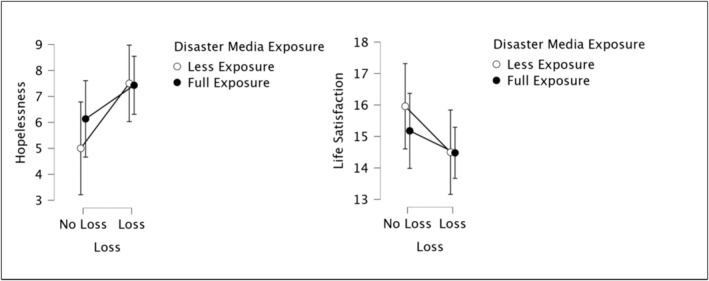
Effects of DME and loss on hopelessness and life satisfaction.

Loss and level of hopelessness were significantly associated (*F*_1,210_ = 4.58, *p* < 05). As such, people who had a loss in the earthquakes had higher levels of hopelessness (*M* = 7.45; *SD* = 5.39) as compared to others who did not (*M* = 5.74; *SD* = 4.67). There was no significant relationship between DME and level of hopelessness (*F*_1,21_ = 0.24, *p* > 0.05). In addition, no interaction effect was found between DME and loss (*F*_1,210_ = 0.49, *p* > 0.05) (see Figure 3).

#### 3.1.7 Life Satisfaction

Life satisfaction was not significantly related to either DME (*F*_1,210_ = 0.07 *p* > 0.05) or loss (*F*_1,210_ = 1.56, *p* > 0.05). There was no interaction effect between DME and loss (*F*_1,210_ = 0.25, *p* = > 0.05) (see Figure 3).

### 3.2 Temporal‐Psychological Processes

#### 3.2.1 DD

**FIGURE 4:**
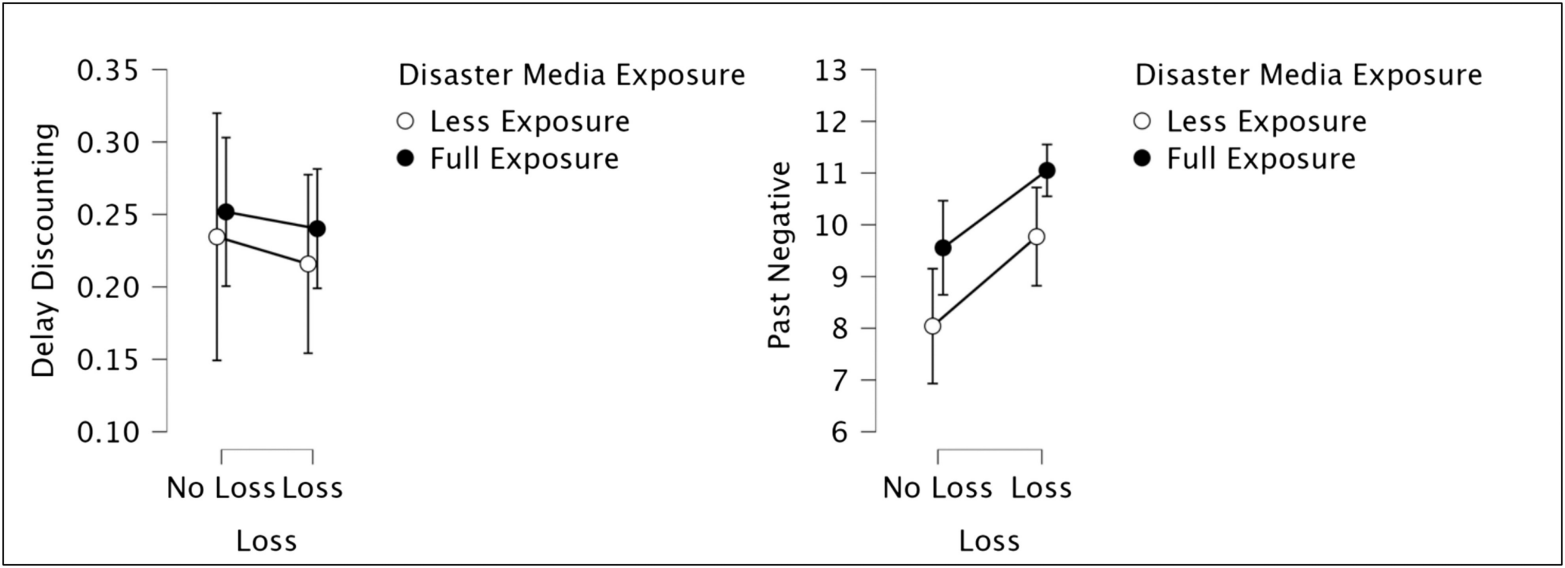
Effects of DME and loss on past‐negative time perspective and delay discounting.

DD did not show a significant relationship with either loss (*F*_1,210_ = 0.94, *p* > 0.05) or disaster media (*F*_1,210_ = 0.08, *p* > 0.05). There was no interaction effect between disaster media and loss (*F*_1,210_ = 0.05, *p* > 0.05) (see Figure 4).

#### 3.2.2 Time Perspectives

Five dimensions of the time perspectives were taken into ANCOVA. Only the past‐negative showed a significant relationship with both of the independent variables. Loss and past‐negative were significantly related (*F*_1,210_ = 10.14, *p* < 0.01): Individuals who had lost someone in the earthquakes had more tendency to have a past‐negative (*M* = 10.63; *SD* = 0.2.83) than others who did not (*M* = 9.03; *SD* = 0.2.96). Furthermore, DME and past‐negative were also significantly related. In particular, participants who had been exposed to disaster media fully were more prone to have a past‐negative (*M* = 10.58; *SD* = 2.75) than others who had been exposed less (*M* = 9.19; *SD* = 3.16) (*F*_1, 210_ = 7.91, *p* < 0.01). There was no interaction effect between loss and DME (*F*_1,210_ = 0.02 *p* = 0.88) (see Figure 4).

## 4 Discussion

The current study aimed to explore the impact of the indirect exposure to the 2023 Turkey earthquakes on the psychological states and temporal‐psychological processes, investigating particularly the effect of losing someone and being exposed to disaster media repeatedly. Findings demonstrated two main crucial findings. Firstly, individuals who have lost someone in the earthquake—even if they did not experience the earthquake directly—showed higher levels of earthquake‐related trauma severity, anxiety, hopelessness and a more pronounced past‐negative time perspective, as compared with those who did not lose someone. Secondly, individuals who were fully exposed to disaster media had higher levels of earthquake‐related trauma, PTSD symptoms, depression, anxiety, stress and a more pronounced past‐negative time perspective than others who were less exposed. Importantly, the DME had an amplifying negative effect on the impact of losing someone's well‐being by increasing the levels of earthquake traumatization. Importantly, the present study was the first study to explore the effect of indirect trauma and DME on a variety of temporal‐psychological processes, showing an enhanced focus on past‐negative time perspective in individuals who lost someone in the earthquakes and that were repetitively exposed to disaster media.

In contrast to the studies that failed to find a relationship between loss and traumatization (Goenjian et al. 2009), current findings were supported by other authors (Dell'Osso et al. 2011). After a traumatic experience, the severity of traumatization was higher in individuals who had a bereavement due to a loss than in others who did not. Losing someone in an earthquake is such a strong experience that even for people who did not experience the earthquakes directly, it was enough to create severe earthquake traumatization. Therefore, it is important to assess the level of traumatization after the earthquakes to identify the ones who are at risk of developing PTSD. Likewise, full exposure to disaster media was related to more severe levels of earthquake trauma, in line with the literature (Hall et al. 2019). Interestingly, the severity of traumatization in case of losing someone was modulated by the frequency of DME: People who have lost someone in the earthquakes and who were also exposed to disaster media all day long showed higher levels of traumatization as compared with individuals who also had a loss but were less exposed to media. This crucial finding proves the impact of media on amplifying the level of traumatization on individuals with the same traumatic experience of losing someone.

Furthermore, individuals who were fully exposed to disaster media had more PTSD symptoms than others who were less exposed. This result is new and remarkable as it demonstrates that even in the absence of a direct experience, repetitive DME was strictly associated with the development of PTSD symptoms. According to DSM‐V‐TR guidelines, repeated exposure to the event has been only considered as a trauma in case it is profession‐related (APA 2022). However, the current study found that repeated DME was associated with elevated PTSD symptoms not only in professions related to disasters but also in general populations. Thus, DME, regardless of profession, should be taken into account while identifying trauma.

In line with the previous research (Pfefferbaum, Nitiéma, and Newman 2021), individuals who have been exposed to disaster media continuously had higher levels of depressive symptoms than others who have been exposed less. Hence, it is essential to inform people about the outcomes of DME to avoid the risk of depression.

One of the current findings of the study suggested that having a loss was not related to higher levels of depression, which was in contrast to the existing literature reporting that loss can be a risk factor for presenting higher levels of depression (Gao et al. 2019). The reason could be the common thought patterns in depression that are usually self‐related, for example, self‐worthlessness (Hoffmann et al. 2016). However, in the case of a loss, the feeling of lack of control over things (Massazza, Brewin, and Joffe 2021) is more pronounced rather than self‐related patterns encountered in depression. Consistent with the previous literature (Fiorenzato et al. 2021), as DME increases, anxiety symptoms increase as well. This could be attributed to the overwhelming presence of distressing content in the media during disaster periods. Another vital finding was that people who lost someone had increased levels of anxiety as compared to others who did not, as confirmed by literature (Shear and Skritskaya 2012). According to these researchers, anxiety is mostly neglected when exploring bereavement; however, the loss of a close one might lead to anxiety disorders. In addition to anxiety and depression, recurrent DME was related to higher levels of stress. The primary reason for this connection may be the perceived emotional strain of the media content, as it often features distressing news stories (Palgi, Shrira, and Hoffman 2017).

Although life satisfaction has been shown to decrease with increased trauma exposure (Krause 2004), no study has yet examined the impact of losing someone to trauma and its relation to life satisfaction. Findings from the current sample and design revealed that neither experiencing a loss nor DME affected life satisfaction. Future research should investigate changes in life satisfaction using larger samples with diverse exposure types. Importantly, high levels of hopelessness were associated with losing someone in the earthquake. According to the hopelessness theory of depression (Abramson, Metalsky, and Alloy 1989), hopelessness intensifies after negative experiences, shifting thought processes towards distress and negativity.

Therefore, bereavement might lead to elevated hopelessness. Aiming to change the level of hopelessness can be a crucial way to decrease the impact of earthquakes.

The findings on the temporal cognitive and psychological processes showed that DD was not affected by different types of earthquake exposure. The primary explanation was the fact that participants of the current study were composed of people who did not directly experience earthquakes. Scholars claimed that steeper discounting was only observed in individuals who directly experienced it (Li et al. 2012); therefore, the findings of the current sample suggest that secondary types of exposures might not be powerful enough to influence DD. Moreover, the current sample size might be not large enough to make smaller effects emerge. Hence, non‐significant results (e.g., DD tendency) may not imply a lack of relationship between variables but rather an underpowered finding. Future studies should aim for larger sample sizes to investigate post‐traumatic situations with the current variables. Secondly, the extreme inflation rates in Turkey might be impactful on the temporal decisions made; reported as 55.18% and 43.68% for the month that the earthquake happened and when the present study was conducted, respectively (Turkish Statistical Institute). This economic situation might lead to a preference for smaller but sooner gains due to the inevitable reduction of the value of the Turkish lira.

Interestingly, losing someone in the earthquakes and full DME was associated with an increased tendency to have an overemphasized past‐negative time perspective, which was in line with the literature on trauma and time perspectives (Micillo et al. 2022; Senyk et al. 2022). Individuals who have lost someone or who were exposed to disaster media all day long tend to overvalue the negative memories of the past.

The first limitation of the present study was the classification of the sample and the disparity in the numerosity among the different groups. The use of two categories for the ‘loss variable’ did not allow us to explore whether the level of traumatization could be influenced by the closeness of the relationship (e.g., losing a spouse or child vs. losing an acquaintance). Despite this limitation, the present study provided important insights in revealing that even indirect exposure, such as loss of someone or repetitive exposure to disaster media, can lead to post‐traumatic responses. Secondly, the sample size was small for the number of variables investigated. This study was exploratory in nature, aiming to assess various psychological and cognitive variables to understand the aftermath of a disaster from multiple perspectives. However, the sample size was relatively small for the number of variables measured. Hence, the findings may be underpowered. A future recommendation would be to explore the aftermath of trauma in larger samples. Thirdly, scores on the continuous disaster media exposure variable were not equally distributed (6‐point Likert scale) that created a problem while analysing the impact of different levels of media exposure as a continuous variable. Frequencies table (see Supporting Information) showed that almost the half of the sample was accumulated in the score 6, corresponding to media exposure throughout all day long, whereas the rest of the scores were not enough to compare individually (1–5). This accumulation of responses would not allow for the analysis it as a continuous variable; hence, combining the responses that represent less frequent media exposure (1–5) and comparing it with the full exposure (6) was the optimal way. Acknowledging this limitation in our methodology, future studies with a sample with an equal distribution of media exposure are recommended while investigating post‐trauma.

Taking all the findings together, earthquake trauma is closely related to PTSD, anxiety, stress, depression, hopelessness and past‐negative orientation even in individuals who did not experience it directly. In the case of massive natural disasters, it is crucial to assess the post‐traumatic situation in all countries. Indeed, not only the ones who directly experienced the event but also people who were exposed to the earthquakes at different degrees seem to be at risk of developing psychopathology and thus require psychological aid. In countries such as Turkey, where devastating disasters are common, the post‐traumatic assessment should be applied to everyone and aim to qualify for preventive mental health services. Importantly, easily accessible psychological help services are important following an earthquake trauma; therefore, governments should prioritize psychological recovery as much as physical recovery. A novel finding is also the role of the media in disturbing psychological states, amplifying the effect of losing someone on trauma. Government and psychoeducational organizations should make individuals aware of this relationship and discourage the dysfunctional continuous use of trauma‐related media. Lastly, in the current technological era, traumatic scenes are brought directly into households, potentially affecting everyone. Therefore, future editions of the DSM might consider the impact of media on psychological well‐being during disasters when defining trauma.

## Ethics Statement

The study was conducted in accordance with the Declaration of Helsinki and approved by the Ethical Committee of Padua for psychological research.

## Conflicts of Interest

The authors declare no conflicts of interest.

## Supporting information


**Data S1** Supporting Information

## Data Availability

Data are available upon request.
