# Supplementary material for: Impact of Indirect Trauma and Disaster Media Exposure on Psychological States and Temporal Processes: The Case of 2023 Turkey Earthquakes
Source: Clin Psychol Psychother. 2024 Nov 21;31(6):e70008. doi: 10.1002/cpp.70008 (PMC11579812; doi:10.1002/cpp.70008)
Supplement: Supplementary file 1 — Data S1 Supporting Information [file CPP-31-e70008-s001.docx]

**Supplementary Documents**

Methods

*Scale That Determines the Level of Trauma after the Earthquake*

After an earthquake in 2011 in Turkey, scholars developed a culturally suitable instrument that screens the extent of the trauma after the earthquake for the Turkish population (Tanhan & Kayri, 2013). Scholars aimed to create a questionnaire specifically for the post-traumatic symptoms after an earthquake in Turkey, which matches the present study’s goals. The scale was developed in Turkish and had 20 items where each item had 5-point-Likert options (1 = I do not agree at all, 2 = I agree little, 3 = I agree at the medium level, 4 = I agree very much, 5 = I completely agree). Some sample items were “Because I am afraid that there will be an earthquake, I can’t enter indoor places.” and “After what I have been through, my desire to live has decreased.” The score was calculated by summing up all the responses where the lowest score can be 20 which corresponds to the lowest level of trauma whereas the highest score can be 100 which corresponds to the highest level. Exploratory Factor Analysis (EFA) showed the five factors of the scale as behavioral problems (α = 0.64), emotive limitedness (α = 0.75), affective (α = 0.61), cognitive structures (α = 0.68), and sleep problems (α = 0.70); with their Cronbach alpha levels, respectively. In this study, the internal consistency of the scale was α = 0.92 whereas in the adaptation of the scale, it was α = .87 (Tanhan & Kayri, 2013).

*PTSD Checklist for Diagnostic and Statistical Manual of Mental Disorders, Fifth Edition (PCL-5)*

PCL-5 is designed for assessing the severity of the traumatic symptoms after a traumatic experience mapping onto DSM-V symptoms (APA, 2013). The first version consisted of 17 items (Weathers et al., 1993) while the final version of PCL-5 was expanded to 20 items (Weathers et al., 2013).

Questions in the checklist concerning the past month's experiences with a 5-point-Likert scale (0 = not at all bothersome, 1 = very little bothersome, 2 = moderately bothersome, 3 = considerably bothersome, 4 = extremely bothersome). “In the past month, how much were you bothered by: ‘Having strong physical reactions when something reminded you of the stressful experience (for example, heart pounding, trouble breathing, sweating)?’ and ‘Trouble remembering important parts of the stressful experience?” are some example items of the scale. The total score is calculated by summing up all the responses, thus, the highest score is 80 indicating a high level of post-traumatic symptoms, whereas the lowest is 0 indicating a low level. In DSM-5, PTSD symptoms are classified into four categories that are the four factors of PCL-5: re-experiencing the memories related to the event, recurrent dreams about the event (criteria B), avoidance from the reminders, memories, places of the traumatic event (criteria C), negative alterations such as self-blame, having negative emotions for prolonged times, negative cognition about self/others/world (criteria D), and hyperarousal, hypervigilance, increased reactivity (criteria E).

The Turkish reliability and validation study of the PCL-5 has been done by Boysan and colleagues (2017) which has been proven to distinguish PTSD patients from both the healthy control group and patients with depression. The cut-off score recommended for the community samples is 48, which had been used in the present study. When reporting the internal consistency of the Turkish adaptation of PCL-5, researchers used a composite reliability coefficient. This method measures the reliability of the multi-item scales, e.g., PCL-5. The first value represents the reliability of the control group while the second value represents the reliability of the clinical group, in this case, it was PTSD patients. For the Turkish PCL-5, the coefficients were reported accordingly: criterion B (re-experiencing) was .79 and .92, criterion C (avoidance) was .73 and .91, criterion D (negative alterations) was .85 and .90, and criterion E (hyperarousal) was .81 and .88. Lastly, for the overall scale it was reported as .94. and .97. The reliability score was α = .97 while for the present study, the Cronbach’s alpha was .94.

*Depression, Stress, and Anxiety Scale (DASS-21)*

The Turkish adaptation of the DASS-21 by Yılmaz, Boz, and Arslan (2017) was used. DASS-21 consists of 21 items; 7 items per each of the three factors depression, stress, and anxiety. The reliability of the total scale was .93 (Henry & Crawford, 2005). The original scale has a high internal consistency (.80 for anxiety, .81 for depression, and .75 for stress). In the current study, Cronbach’s alpha for the overall scale was .94; and for the subscales they were .91, .83, .89, respectively. The Turkish adaptation for the scale is a combination of 3 self-report questionnaires where each of which had 7 items, 21 in total. The rating was based on a 4-point-Likert scale as 0 “Did not apply to me at all”, 1 “Applied to me to some degree, or some of the time”, 2 “Applied to me a considerable degree, or a good part of the time”, 3 “Applied to me very much, or most of the time”. Some items on the scale are listed: “I was unable to become enthusiastic about anything.”, “I was worried about situations in which I might panic and make a fool of myself”, and “I couldn’t seem to experience any positive feeling at all.” After the Confirmatory Factor Analysis, they reported that the Turkish version of DASS-21 has a high internal consistency with the following Cronbach alpha levels; .80 for anxiety, .81 for depression, and .75 for stress (Yılmaz, Boz, and Arslan, 2017).

*Satisfaction with Life Scale*

Life satisfaction is defined as “a global assessment of a person’s quality of life according to his chosen criteria” (Shin & Johnson, 1978), hence it is a subjective well-being judgment (Diener et al.,1985). The scale is a short self-report with 5 items that form one factor (Diener et al.,1985) The scale aims to quantify how much satisfied are individuals with their lives with a 7-point-Likert rating. The Turkish reliability and validity study of the Satisfaction with Life Scale was done by Dağlı and Baysal (2016) and found a high internal consistency (Cronbach alpha = .88), and high test-retest reliability (.97) testing again after two weeks of administration. Additionally, the Pearson product-moment correlation coefficient showed that the Turkish scale and English scale were correlated (*p* = .92) which proves that the scale is a reliable and valid measure of life satisfaction for the Turkish population (Dağlı & Baysal, 2016). During the adaptation studies of scale into Turkish culture, the options in the 7-point Likert were perceived as close to each other by the Turkish participants. To have an easily understandable scale and prevent confusion, scholars decreased the number of options to a 5-point Likert (1 = Strongly disagree, 2 = Slightly disagree, 3 = Agree moderately, 4 = Slightly agree, 5 = Strongly agree). To calculate the overall score, each point in the options is coded based on their values, ranging from 5, the lowest score, to 25, the highest score. “The conditions of my life are excellent.” is an example item.

*Beck Hopelessness Scale*

Hopelessness refers to “a lack of enthusiasm, a motivational tendency to give up, and a dearth of positive expectancies.” (Beck, 1974). The original Beck Hopelessness Scale was developed to quantify the level of hopelessness of individuals (Beck et al., 1974). To assess the hopelessness level, the Turkish version of the Beck Hopelessness Scale (Seber et al., 1993) was used. The scale consisted of 20 self-report questions divided into 11 normal questions such as “I might as well give up because there is nothing I can do about making things better for myself.” and “I never get what I want, so it is foolish to want anything.”, and 9 reverse coded questions such as “My past experiences have prepared me well for the future.” and “I have great faith in future.” The answer options were either “yes” or “no”. The total score of the scale is called the hopelessness score and is determined by giving 1 point for each “yes” and 0 points for each “no”. Items 2, 4, 7, 9, 11, 12, 14, 16, 18, and 20 were positively coded while items 1, 3, 5, 6, 8, 10, 13,15, and 19 were reverse coded. The score on the scale ranges from 0 to 20. There are 3 factors in the scale emotional, motivational, and cognitive. (Seber et al., 1993). The Cronbach alpha of the original scale was .93 while the Turkish adaptation and reliability study had a high internal validity, (.86).

*Delay Discounting Task (DDT)*

Following the questionnaires, participants were informed that they were going to be directed to another platform to complete another task. DDT is a measure of future-oriented behavior for decision-making. In this decision-making task, participants were asked to choose between two hypothetical monetary alternatives: A smaller but sooner amount (e.g., 400.000 ₺, now) versus a larger but later amount (e.g., 800.000 ₺ in 1 year). To create different time points, six delays as 1 month, 6 months, 1 year, 3 years, 5 years, and 10 years were used. In DDT, the amount that participants tend to choose equally either a smaller but sooner (SS) amount or a larger but later (LL) amount is called the indifference point. The indifference point represents an unshown immediate amount, that does not change the subjective value of the reward for the participants. To measure the indifference point, participants were required to make five choices for each delay. The immediate amount started at 400.000 ₺, which was modulated according to the participants’ previous preferences (increased versus decreased amount), while the delayed amount was fixed at 800.000 ₺. Six delays were given in a fixed order for all participants (Curtis et al., 2018). Money amounts were presented with the symbol of Turkish Lira (₺). OpenSesame v.3.3.6 was used to design the task and it was run online through JATOS (Lange, Kühnhausen, and Filevich, 2015) with the help of OSWeb v.1.3.11 extension (Mathôt et al., 2012). Based on the indifference points at each time delay, the area under the curve (AUC) was calculated for each one of the delays to assess the subjective value using a quantitative approach of delay discounting. AUC ranges from 0 (high discount rate) to 1 (low discount rate). Even though DDT is based on hypothetical rewards, it gives crucial information regarding temporal decision-making.

*Zimbardo Time Perspectives Inventory (ZPTI-15)*

Time perspective is a term explaining how individuals unconsciously project themselves around time (past, present, and future) based on the links and values they have created between experiences and time periods (Zimbardo & Boyd, 1999). The scale has 5 dimensions as follows: Past-Positive, Past-Negative, Present-Hedonic, Present-Fatalistic, and Future. Past-Positive is associated with the positive and peaceful memories and thoughts of the past, on the contrary, Past-Negative represents the cold and unpleasant view of the past. The third dimension, Present-Hedonistic, is related to focusing on the immediately available pleasures while not taking the future into account at all, which is often linked with risk-taking behaviors. Present-Fatalistic, on the other hand, reflects a negative, ‘leaving everything to fate’, and hopeless perspective about the future. It is linked with less future-oriented thinking. Lastly, the Future dimension is represented when the actions are decided based on their future outcomes.

Later on, a short version of the scale was developed with 15 items (Kostal et al., 2016).

Kocayörük and Şimsek (2020) created the Turkish version of the ZPTI-15 short form with a 5-point Likert scale. (1 = Very uncharacteristic, 2 = Uncharacteristic, 3 = Neutral, 4 = Characteristic, 5 = Very characteristic). The Turkish adaptation of ZPTI-15 is proven to be valid and reliable according to the Cronbach alpha level ranging from .65 to .78. Example items were listed as follows: “It gives me pleasure to think about my past.” for Past Positive; “I think about the bad things that have happened to me in the past.” for Past Negative, “It is important to put excitement in my life.” for Present Hedonistic, “When I want to achieve something, I set goals and consider specific means for reaching those goals.” for Future (Kocayörük& Şimşek, 2020).

Discussion

| *Frequencies for Continuous Disaster Media Variable* | | | | | | | | | |
| --- | --- | --- | --- | --- | --- | --- | --- | --- | --- |
| Disaster media(continuous) | | Frequency | | Percent | | Valid Percent | | Cumulative Percent | |
| 1 |  | 11 |  | 5.116 |  | 5.140 |  | 5.140 |  |
| 3 |  | 7 |  | 3.256 |  | 3.271 |  | 8.411 |  |
| 4 |  | 7 |  | 3.256 |  | 3.271 |  | 11.682 |  |
| 5 |  | 47 |  | 21.860 |  | 21.963 |  | 33.645 |  |
| 6 |  | 142 |  | 66.047 |  | 66.355 |  | 100.000 |  |
| Missing |  | 1 |  | 0.465 |  |  |  |  |  |
| Total |  | 215 |  | 100.000 |  |  |  |  |  |
|  | | | | | | | | | |
